# Esophagojejunostomy Feeding Tube Placement in 5 Dogs with Pancreatitis and Anorexia

**DOI:** 10.1155/2014/197294

**Published:** 2014-03-06

**Authors:** Forrest Cummings, Catherine A. Daley

**Affiliations:** Metropolitan Veterinary Specialists, 11800 Capital Way, Louisville, KY 40299, USA

## Abstract

Enteral feeding tube placement has been described in veterinary medicine for several years. Indications include oral, esophageal, gastrointestinal, pancreatic, hepatic, and neurologic diseases. In this paper, endoscopically assisted placement of an esophagojejunostomy (EJ) feeding tube in dogs with pancreatitis and prolonged anorexia is described. To the author's knowledge there are no published reports of this procedure. Esophagojejunostomy feeding tubes provide an alternative to other forms of postgastric feeding tube placement (e.g., nasojejunal, gastrojejunostomy, and jejunostomy tubes) without the associated complications of patient discomfort, sneezing, epistaxis, and peritonitis. Tube occlusion, transient vomiting and loose stool were the most commonly reported complications.

## 1. Introduction

Enteral tube feeding has been used in veterinary medicine for a number of years. Oral, esophageal, gastrointestinal, pancreatic, hepatic, and neurologic diseases that would otherwise result in anorexia and malnutrition are all indications for enteral feeding tubes [1–3]. Several techniques have been used to describe the placement of feeding tubes, the frequency of feeding, and potential complications associated with each procedure. Nasoesophageal, pharyngostomy, esophagostomy, gastrostomy, jejunostomy, gastrojejunostomy, and nasojejunal feeding tube placement have all been described in the literature. Feeding tubes may also be placed without endoscopic assistance for percutaneous gastrostomies [4], as well as with nasogastric, pharyngostomy, and esophagostomy tube placement. Endoscopic, fluoroscopic, laparoscopic, or surgical assistance has been used for gastric and jejunal feeding tube placement [1, 5, 6]. Reported complications of feeding tube placement include patient discomfort, premature tube removal by the patient, epistaxis, stoma site infection, peritonitis, tube occlusion, vomiting, diarrhea, abdominal pain, and retrograde tube displacement. The purpose of this study was to describe an alternative for postgastric enteral feeding tube placement and to assess the advantages and potential complications of its use.

## 2. Materials and Methods

Five client-owned dogs had an esophagojejunostomy (EJ) tube placed using a weighted-tipped 8-Fr feeding tube. ^1^ All dogs presented to our hospital for evaluation of vomiting and anorexia of unknown etiology. Duration of clinical signs ranged from 2 to 15 days prior to evaluation at the authors' facility. Case 1 was an 11-year-old, male neutered Yorkshire Terrier. Weight and body condition score at the time of admission were 10 pounds and 3 out of 9, respectively. Case 2 was a 12-pound, 11-year-old, female spayed Yorkshire Terrier mix with a BCS of 7/9. Case 3 was a 14-pound, 7-year-old, female spayed Cocker Spaniel with a BCS of 3/9. Case 4 was an 18.6-pound, 2-year-old, female intact Brussels Griffon with a BCS of 5/9. Case 5 was a 32-pound, 4-year-old, female spayed Terrier mix with a BCS of 4/9. A complete blood count, biochemical profile, urinalysis, abdominal radiographs, and abdominal ultrasound were performed in all dogs prior to EJ feeding tube placement. In addition, pancreatic lipase immunoreactivity assays and serum cobalamin and folate levels were performed in Cases 1–3. In addition to having a gastroduodenoscopy, gastric and duodenal biopsies were performed at the time of EJ feeding tube placement in Cases 3–5. All dogs had an EJ feeding tube placed within 24 hours of admission. Pancreatitis was the cause for vomiting and anorexia in Cases 1, 2, 4, and 5. Pancreatitis was diagnosed by identifying severely elevated lipase and amylase after a 12-hour fast, ultrasonographic changes, and clinical signs. In addition, an elevated serum PLI was noted in Cases 1 and 2. The cause for vomiting and anorexia noted in Case 3 was attributed to primary diffuse inflammatory bowel disease based on the histologic findings of lymphoplasmacytic gastroenteritis.* Helicobacter-like *organisms were also noted on gastric histopathology. In addition, serum cobalamin levels were also severely decreased, indicating distal small intestinal disease. In contrast, Case 3 had normal serum biochemical parameters, no abnormal pancreatic changes on ultrasound, and normal serum PLI. In addition, Case 3 had an elevated resting cortisol, making hypoadrenocorticism unlikely.

Supportive therapy for the dogs diagnosed with pancreatitis included intravenous crystalloid therapy, empiric antibiotics, antiemetics, and pain management. All dogs received IV Lactated Ringer's Solution ^2^ with potassium chloride ^3^ supplementation to achieve a concentration of 20 mEq KCl/L at a rate of 45–60 mL/kg/day.  Enrofloxacin ^4^ 5 mg/kg IV Q 24 H and ampicillin ^5^ 22 mg/kg IV Q 8 H or cefazolin ^6^ 22 mg/kg IV Q 12 H was administered.  Metoclopramide ^7^ 0.3 mg/kg IV Q 8 H or 1.1 mg/kg/day IV as a constant rate infusion, maropitant ^8^ 1 mg/kg SC Q 24 H, or ondansetron ^9^ 0.1–0.5 mg/kg IV Q 12–24 H were used to control vomiting.  Butorphanol ^10^ 0.1 mg/kg IV Q 4–6 H IV or buprenorphine ^11^ 0.02 mg/kg IV Q 6 H was administered for pain control.  Famotidine ^12^ 0.5 mg IV Q 24 H was used to decrease gastric acid production.

Case 3 was treated with IV fluid therapy as previously prescribed. Anthelmintic therapy with fenbendazole ^13^ 50 mg/kg PO Q 24 H for 3 days was initiated. The dose was repeated in 3 weeks.  Amoxicillin ^14^ 20 mg/kg PO Q 12 H, sucralfate ^15^ 250 mg PO Q 8 H, and metronidazole ^16^ 10 mg/kg PO Q 12 H were administered for 3 weeks to treat the* Helicobacter-like *organisms.  Prednisone ^17^ 1.5 mg/kg PO Q 24 H was used to manage the inflammatory bowel disease. Diet modification using a novel protein was postponed until the vomiting had ceased and the appetite had returned. Hospitalization time ranged from 1 to 15 days after EJ feeding tube placement with a median of 6 days.

**Figure 1:**
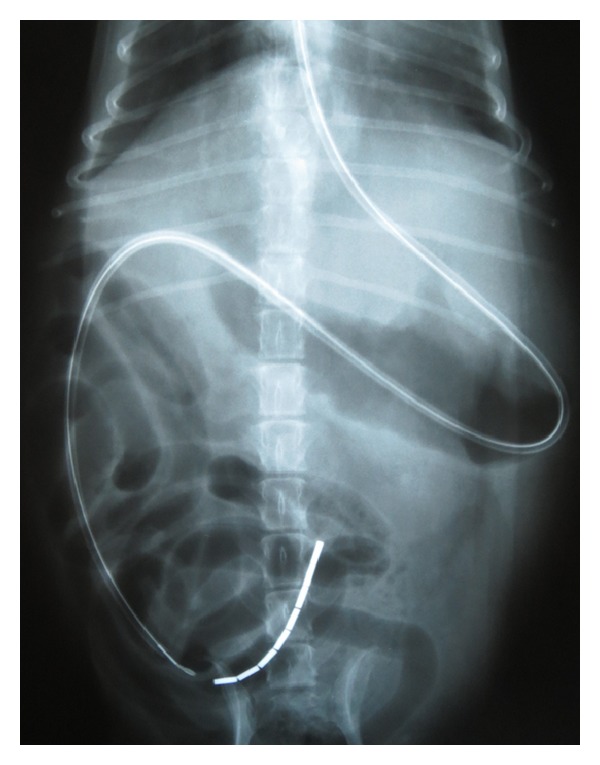


**Figure 2:**
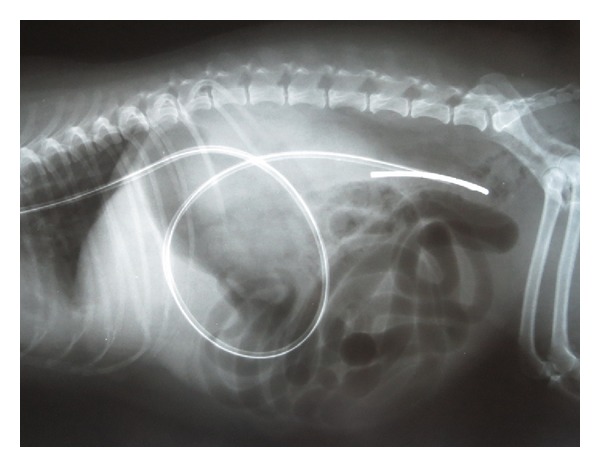


Immediately following the gastroduodenoscopy, the dogs were positioned in right lateral recumbency for placement of the EJ feeding tube. A 5 cm × 5 cm area on the left lateral cervical region was clipped and aseptically prepared [7]. Curved hemostats were inserted orally into the esophagus and directed laterally to allow for external access through the esophagus. The jugular vein was occluded at the thoracic inlet so that it would distend and be avoided when penetrating the skin. Using a number 11 scalpel blade, the skin was then incised over the hemostats to the level of the esophagus. Once the hemostats were exteriorized, they were used to grasp the distal end of the feeding tube which was pulled into the esophagus and out of the mouth. The feeding tube was then reinserted into the mouth and directed into the stomach. The dog was then placed in left lateral recumbency. The endoscope was inserted through the oropharynx and into the stomach to locate the feeding tube. Once identified, the EJ tube was secured and directed to the pylorus using grasping forceps. Insertion of the tube into the duodenum was achieved by repeatedly grasping and advancing the tube with the grasping forceps. Once the tube was inserted into the duodenum approximately 5 cm, it was advanced further by slowly pushing at the level of the esophagus for an additional 30 cm. If coiling of the feeding tube was noted in the stomach, the tube was pulled at the level of the esophagus, while it was simultaneously grasped with the forceps at the level of the pylorus to prevent retrograde movement within the small intestines. Ventrodorsal and lateral abdominal radiographs were taken to confirm placement in the proximal jejunum (Figures 1 and 2).

After correct placement was confirmed, the stylet was removed and tube patency was checked using warm water. The EJ tube was then secured at the skin with nonabsorbable suture. After securing the tube to the skin, the patency was checked again with water to ensure that the suture was not tied too tightly. A small bandage was placed over the esophagostomy site. An Elizabethan collar was placed on each dog after placement of the EJ tube. The average time for gastroduodenoscopy and EJ tube placement was approximately 50 minutes.

The resting energy requirement (RER) with an illness factor of 1.2 was used to calculate the metabolic energy requirement (MER) for each patient using the following equation: MER = (30 ∗ body wt kg + 70) ∗ 1.2. A liquid diet ^18^ was initiated via the tube within 8–24 hours after EJ feeding tube placement. For the first 24 hours, all patients were fed 25–33% of their daily MER, divided into 3-4 feedings or as a constant rate infusion (CRI). The feeding amounts were to be increased until each dog was receiving its calculated daily energy requirement within 4-5 days. Other than the fenbendazole, all medications were given parenterally to avoid occlusion of the feeding tube. Patency was to be maintained by flushing the tube with warm water every 4–6 hours. Three to five milliliters of carbonated water was placed into the feeding tube if saline failed to remove any obstruction. The stoma site was initially evaluated after 24 hours, after 3–5 days, and weekly thereafter. In addition, all patients had their weight monitored weekly.

Case 1 was discharged into the care of its primary veterinarian 24 hours after EJ feeding tube placement due to ongoing cost of therapy. He was subsequently euthanized after 3 days, while still under veterinary care, due to complications not associated with pancreatitis or the EJ feeding tube placement. Persistent vomiting and progressive lethargy were noted despite supportive care. Neither the owner nor the primary veterinarian reported any complications with the feeding tube. Neither a weight nor BCS was obtained from this patient at the time of death.

Case 2 was discharged from the hospital at the owner's request after 7 days. The feeding tube was functional, the dog had started to regain an appetite, and clinical signs associated with pancreatitis were improving. The owner supplemented oral feeding with boluses of CliniCare via the EJ tube. The feeding tube was subsequently removed after 11 days because all signs of pancreatitis had resolved. Its weight at the time of tube removal was 11.5 pounds (6/9).

Case 3 was discharged from the hospital after 3 days at the owner's request. The vomiting had resolved; however, the dog remained anorexic and continued to lose weight. CliniCare was changed from a CRI to bolus feeding via the feeding tube 3–5 times daily so the owner could continue therapy at home. In addition, Prescription Hill's I/D was prescribed to encourage normal enteral feeding. The EJ feeding tube remained functional for 45 days before removal. Mild cellulitis developed at the stoma site 22 days after EJ tube placement. The wound was cleaned with chlorhexidine solution and the bandage was replaced. The wound was reassessed after one week and it had healed. The owner elected to continue to administer CliniCare via the EJ tube with bolus feeding, as the hyporexia continued. The weight at the time of tube removal was 12 pounds (BCS 2/9). The owner reported intermittent tube occlusion, infrequent vomiting, and soft stool during the time that the tube was in place. There were no other complications reported at the stoma site.

Case 4 remained hospitalized for 6 days. The major complication noted during hospitalization was that the CRI would occlude frequently due to excessive patient movement. The CRI was discontinued after 12 hours and bolus feeding via the EJ tube was initiated. Vomiting was noted each day but the frequency lessened daily. The owner did not utilize the EJ tube at home because the dog was eating. Although the tube was not being used to administer food, the owners were unable to successfully maintain patency with frequent flushing. The tube was removed after 13 days. Weight at the time of removal was 18.75 pounds (BCS 5/9).

Case 5 remained hospitalized for 11 days. Vomiting occurred; however, the frequency of vomiting lessened each day. The CRI of CliniCare was continued. The vomiting resolved after 5 days. The dog began to regain interest in food after 8 days and the use of the feeding tube was discontinued on day 10. The EJ feeding tube was removed after 15 days. The owner did not report any complications after discharge. Weight at the time of feeding tube removal was 29.5 pounds (BCS 3/9).

After patient discharge, complications reported were tube occlusion (2/5), infrequent vomiting (4/5), stoma site infection (1/5), and soft stool (1/5). All owners reported that their dog's activity and comfort level appeared to normalize after discharge. Only one dog (Case 3) had abdominal radiographs repeated in order to assess whether retrograde tube placement had occurred. The radiograph confirmed correct placement after 45 days.

## 3. Discussion

Endoscopically assisted EJ feeding tube placement provides an alternative in dogs requiring postgastric enteral nutrition without encountering complications seen with other techniques. Nasally placed enteral feeding tubes may result in epistaxis and sneezing. Furthermore, surgically and laparoscopic-assisted enteral feeding tubes require a laparotomy and gastrotomy or enterotomy, thus predisposing the patient to peritonitis if there is dehiscence at the surgical site. Although direct placement of the feeding tube into the gastrointestinal tract immediately confirms the correct location, radiography also proved to be a quick method to confirm appropriate feeding tube placement. An advantage to endoscopically assisted placement with radiographic confirmation compared to fluoroscopic assistance is reduced radiation exposure to the patient and personnel. Due to the small number of dogs in this study, an accurate assessment of chronic use could not be determined. However, in Case 3, the EJ feeding tube was still in the appropriate location within the jejunum and maintained patency until the time of removal at day 45. Furthermore, serial radiographs should be taken to assess retrograde tube migration with chronic as well as short-term use. In this study, the vomiting was most likely secondary to the underlying disease process and not from the administration of food or the EJ feeding tube itself. The incidence of vomiting decreased in all dogs despite increasing the volume of food administered. It was the authors' intent to maintain hospitalization until each dog received 100% of its MER via CRI; however, due to the owner's request (Cases 1 and 3) and a fractious behavior (Case 4) this could not be achieved in all patients. It was also the intent to begin tube feeding within 8–12 hours. The primary factor that influenced this was the patient's level of alertness following anesthesia since all patients were experiencing vomiting. Cases 1 and 2 were still sedate 8 hours following anesthesia; therefore, we elected to postpone tube feeding by additional 8–12 hours. The prolonged sedation was attributed to the age of the patient and the deleterious effects of pancreatitis, since Cases 3–5 did not experience the same sedative effects.

Early literature suggests that enteral feeding was performed as a CRI due to the concern that abdominal cramping, vomiting, and diarrhea would develop due to overwhelming the gastrointestinal neural and endocrine systems [8]. However, enteral feeding when fed as a CRI or with intermittent boluses has been found to be well tolerated in dogs [3, 9, 10]. In the present study, intermittent bolus feeding also allowed for patients to be cared for outside of the hospital setting which resulted in shorter hospitalization times and reduced costs to the owners. No dogs in this study suffered from adverse effects of bolus feeding, thus making it a viable option for postgastric feeding. Chronic prednisone administration and a poor body condition score may have contributed to cellulitis in one dog. Glucocorticoids and malnutrition have been shown to impede wound healing and increase the time to develop a mature stoma [11]. Although cellulitis was observed in this dog, complete resolution of the lesion was observed within one week with local antiseptic therapy and without decreasing or temporarily discontinuing the use of glucocorticoids. The vomiting noted in these dogs was likely related to the underlying disease and not a result of the feeding tube since the frequency lessened over time.

Potential complications with enteral feeding tube placement include tube occlusion, vomiting, diarrhea, abdominal pain, tube site infection, retrograde tube migration, and premature removal by the patient. These are best managed with transient decreases in the volume of food administered, antiemetic therapy, proper wound care management, and maintaining an Elizabethan collar. Potential complication of having an EJ feeding tube placed is the predisposition for developing gastroesophageal reflux and esophagitis as a result of traversing the lower esophageal sphincter. Regurgitation and aspiration pneumonia would be expected in patients with esophagitis; however, these problems were not evident in this study. This may be due to the small-bore feeding tube used, thus allowing the esophageal sphincter not to be significantly compromised. In addition, thoracic radiographs should be performed in cases of regurgitation, persistent vomiting, coughing, fever, or abnormal respiratory patterns in order to assess for pneumonia.

## 4. Conclusion

Endoscopically assisted EJ feeding tube placement provides an alternate method for postgastric enteral feeding in dogs. The procedure provides enteral nutrition to critically ill patients without the risk of developing complications noted with other forms of enteral feeding tubes. Esophagojejunostomy feeding tubes may also have the potential to be used in cases of chronic disease; however, more dogs would be required to accurately assess this claim.

## Endnotes

1. Kendall Entriflex Dual Port 8-Fr Feeding Tube with Flow-Through Stylet, Tyco Healthcare Group LP, Mansfield, MA.

2. Lactated Ringer's Injection, Hospira Inc., Lake Forest, IL.

3. Potassium Chloride Concentrate for Injection, Hospira Inc., Lake Forest, IL.

4. Enrofloxacin Injection, Bayer Healthcare LLC., Shawnee Mission, KS.

5. Ampicillin Injection, Putney Inc., Portland, ME.

6. Cefazolin, West-Ward Pharmaceuticals, Eatontown, NJ.

7. Metoclopramide Injection, Baxter Healthcare Corp., Deerfield, IL.

8. Cerenia (Maropitant), Pfizer Animal Health, Florham Park, NY.

9. Ondansetron Injection, Wockhardt USA LLC., Parsippany, NJ.

10. Butorphanol Tartrate Injection, Intervet Inc., Millsboro, DE.

11. Buprenorphine Hydrochloride, Reckitt Benckiser Pharmaceuticals Inc., Richmond, VA.

12. Famotidine Injection, Baxter Healthcare Corp., Deerfield, IL.

13. Fenbendazole Suspension 10%, Intervet Inc., Millsboro, DE.

14. Amoxicillin Capsules, DAVA Pharmaceuticals Inc., Fort Lee, NJ.

15. Sucralfate Tablets, Nostrum Laboratories Inc., Kansas City, MO.

16. Metronidazole Tablets, TEVA Pharmaceuticals, Sellersville, PA.

17. Prednisone Tablets, Qualitest Pharmaceuticals, Huntsville, AL.

18. CliniCare, Abbott Veterinary Diets, North Chicago, IL.
